# 3D‐Analysis of Correlations Between Facial Measurements and Maxillary Anterior Tooth Dimensions Using Integrated Facial and Intraoral Datasets

**DOI:** 10.1111/jerd.70126

**Published:** 2026-02-16

**Authors:** Kathrin Seidel, Philipp Hericks, Basel El‐Sabbagh, Denise Schlee, Pauline Gutmann, Jan‐Frederik Güth

**Affiliations:** ^1^ Department of Prosthetic Dentistry, Center for Dentistry and Oral Medicine (Carolinum) Goethe University Frankfurt Frankfurt Germany; ^2^ Department of Oral Surgery, Implantology and Oral Medicine, Center for Dentistry and Oral Medicine (Carolinum) Goethe University Frankfurt Frankfurt Germany; ^3^ Department of Prosthetic Dentistry LMU University Hospital Munich Germany

**Keywords:** 3D facial scanning, central incisor width, dental proportions, interalar distance, prosthetic rehabilitation, tip‐to‐tip distance

## Abstract

**Objective:**

This study aimed to evaluate associations between facial measurements and maxillary anterior tooth dimensions using integrated three‐dimensional (3D) facial and intraoral datasets.

**Materials and Methods:**

Eighty‐one participants underwent facial and intraoral 3D scanning. Intraoral parameters included mesiodistal (MeanW1–3) and cervicoincisal dimensions (MeanL1–3) of maxillary anterior teeth and canine tip‐to‐tip distance (TTD). Extraoral parameters comprised interpupillary (IPD), innercanthal (ICD), and interalar distances (IAD). Intra‐examiner reliability was evaluated, and Pearson correlation analyses and proportional ratios were calculated.

**Results:**

Mean measurements were MeanW1 8.76 ± 0.54 mm, MeanL1 10.3 ± 1.01 mm, TTD 34.4 ± 2.21 mm, IAD 33.5 ± 2.89 mm, IPD 61.1 ± 3.29 mm, and ICD 37.0 ± 3.39 mm. IAD showed significant correlations with MeanW1 (*p* = 0.022) and TTD (*p* = 0.004), whereas IPD correlated only with TTD (*p* = 0.006). ICD showed no significant correlations. The TTD/IAD ratio (1.03 ± 0.09) remained consistent across participants. One quarter of TTD approximated the width of a central incisor (MeanW1/TTD = 0.255 ± 0.014).

**Conclusion:**

IAD demonstrated the most consistent association with anterior tooth dimensions. The ratio between TTD and IAD was 1.03.

**Clinical Significance:**

The established ratio between TTD/IAD was approximately 1:1, and one quarter of the TTD corresponded to the width of a central incisor. These measured proportions provide quantitative facial and dental relationships that support the preliminary estimation of anterior tooth dimensions when pre‐extraction records are absent.

**Trial Registration:**

https://drks.de/search/de/trial/DRKS00030166

## 1 Introduction

The selection of maxillary anterior tooth dimensions represents a central aspect in prosthetic rehabilitation, particularly in completely edentulous patients, where esthetic and functional outcomes depend on accurate determination of tooth morphology [1]. Among the various parameters, the mesiodistal width of the anterior teeth has been shown to exert a greater influence on esthetic perception than tooth length [2]. In addition, tooth width remains relatively stable over time, whereas tooth length may be affected by aging, wear, and soft tissue dynamics [3, 4]. Despite the clinical relevance of these dimensions and the availability of numerous indices and selection concepts, no objective and universally accepted reference standard for determining maxillary anterior tooth dimensions has been established [2].

In the absence of a standardized reference, facial proportions have been proposed as potential external guides for anterior tooth selection [5]. Based on the assumption that craniofacial dimensions may reflect dental arch proportions, several extraoral facial measurements, most commonly the interalar distance (IAD), interpupillary distance (IPD), and inner canthal distance (ICD), have been investigated in relation to maxillary anterior tooth dimensions and the maxillary canine tip‐to‐tip distance (TTD). For IAD in particular, different clinical concepts have been described, relating nasal width either to the position of the canine cusp tips or to the distal surfaces of the canines [6, 7, 8]. Consequently, reported conversion factors vary depending on landmark definition, with ratios of approximately 1.03 described when referencing canine cusp tips and higher ratios ranging from 1.26 to 1.31 reported when distal canine surfaces are used [6, 9, 10]. While several studies reported significant correlations between IAD and TTD that support its use in denture tooth selection [3, 6, 8], other investigations observed weak or inconsistent associations [11, 12, 13, 14, 15]. The IPD, considered relatively stable in adulthood [16], has been associated in some studies with the mesiodistal width of the maxillary central incisors or the combined width of the anterior teeth, although reported correlation strengths vary [7, 14, 17, 18]. Similarly, associations between ICD and maxillary anterior tooth width have been described [14, 19, 20]. However, other investigations found no significant relationships for either IPD or ICD [15].

These discrepancies are largely attributable to methodological heterogeneity across studies, including differences in data acquisition techniques, landmark definitions, and the way facial and dental measurements were combined. Earlier investigations relied on a broad spectrum of analog approaches, ranging from direct facial measurements using calipers or rulers to indirect assessments based on two‐dimensional (2D) facial photography. Although direct anthropometric measurements are simple and noninvasive, they are time‐consuming and require a high degree of patient cooperation [6, 19]. Moreover, most studies combined facial measurements obtained from 2D photographs with dental measurements derived from plaster models [3, 5, 8, 10, 13, 18, 21, 22]. In such workflows, facial and dental data were acquired in separate reference systems without spatial correspondence, often using inconsistent landmark definitions across modalities, and were susceptible to projection‐related distortion in photographic analyses. This methodological fragmentation limits the comparability of reported correlations and proportional values across studies, particularly when applied to digitally planned restorative concepts.

Recent advances in three‐dimensional (3D) facial imaging have established digital facial scanning as a valid alternative to conventional measurement techniques. Comparative studies have demonstrated that structured‐light scanners provide measurements comparable to direct anthropometry and allow reproducible identification of facial soft tissue landmarks [23, 24, 25]. When combined with intraoral scanning, 3D facial imaging enables spatial alignment of facial and dental structures within one common coordinate system, eliminating projection‐related distortion and allowing measurements to be archived for repeated analysis [26]. Despite these technical advances, studies investigating the predictive value of 3D facial parameters for anterior tooth dimensions using fully integrated datasets remain scarce. To the authors' knowledge, only one previous investigation has applied a comparable digital approach, focusing on different facial parameters and research objectives [26].

The aim of the present study was to evaluate associations between facial measurements and maxillary anterior tooth dimensions within a fully integrated 3D workflow. Three‐dimensional facial imaging was combined with digital intraoral models to assess mesiodistal and cervicoincisal dimensions of the maxillary anterior teeth, the TTD, and the facial parameters IAD, IPD, and ICD. All measurements were obtained and analyzed within one common 3D coordinate system, enabling spatial alignment of facial and intraoral datasets and standardized evaluation of the consistency and variability of established relationships under digital conditions.

The tested research hypotheses were as follows: (1) Extraoral facial measurements (IAD, IPD, and ICD) show associations with the mesiodistal width of the maxillary central incisors, (2) extraoral facial measurements show associations with the TTD, and (3) proportional relationships between facial measurements and maxillary anterior tooth dimensions are quantifiable within a standardized 3D workflow.

## 2 Materials and Methods

### 2.1 Study Design and Participants

This prospective study was conducted at the Department of Prosthetic Dentistry, Goethe University Frankfurt, Germany. Ethical approval was obtained from the institutional Ethics Committee of Goethe University Frankfurt (approval number: 2022–648), and the study adhered to the Declaration of Helsinki principles. All participants provided written informed consent.

Sample size calculation indicated that 75 participants (±5%) would provide 95% power to detect an effect size of *r* = 0.4 at *α* = 0.05. Ultimately, 81 subjects were recruited. The study sample comprised young, fully dentate adults. Inclusion criteria were age ≥ 18 years, complete permanent dentition, and natural teeth or restorations covering no more than one‐third of the occlusal surface without cuspal coverage. To minimize confounding influences on facial and dental measurements, participants with clinically relevant tooth wear, facial asymmetry, dental abnormalities, temporomandibular or craniocervical disorders, periodontal disease, previous or current orthognathic surgery, or plastic or esthetic surgery affecting facial, ear, or nasal anatomy were excluded. The same cohort was previously analyzed by El‐Sabbagh et al. [27], who investigated the sagittal inclination of the maxillary dentition in relation to facial landmarks. The present study examines the same cohort in relation to a different research question, focusing on parameters not evaluated in the earlier publication.

### 2.2 Data Acquisition

All participants underwent standardized 3D facial and intraoral scanning. Intraoral scans of the maxilla, mandible, and habitual bite were obtained using an intraoral scanner (Primescan 3D, Dentsply Sirona, Bensheim, Germany). Data were exported as stereolithography (STL) files. Facial scans were obtained using a 3D facial scanner (Facehunter, Zirkonzahn, Gais, Italy). Prior to image acquisition, the examiner ensured a relaxed facial expression, natural head position, and habitual occlusion. Five angulated views (0°, +45°, +90°, −45°, −90°) were captured in both closed‐mouth and smiling positions. To enable precise alignment between facial and intraoral datasets, a transfer fork (Zirkonzahn, Gais, Italy) was secured to the maxilla using registration material (Futar D, Kettenbach Dental, Eschenburg, Germany). The fork geometry was digitized with the same intraoral scanner and used to superimpose the maxillary scans onto the facial surfaces via a best‐fit algorithm. Alignment accuracy was verified against the smiling facial scan.

Facial and intraoral datasets were processed using Zirkonzahn scan software and exported as OBJ files. All measurements were performed using GOM Inspect Pro 2022 Service Pack 2 V2.0.1 (Carl Zeiss GOM Metrology GmbH, Braunschweig, Germany).

### 2.3 Intraoral and Extraoral Measurement

**FIGURE 1:**
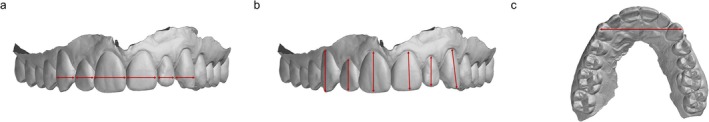
Measurement of (a) mesiodistal widths and (b) cervicoincisal lengths of the maxillary anterior teeth. Width and length measurements are shown for central incisors (MeanW1, MeanL1), lateral incisors (MeanW2, MeanL2), canines (MeanW3, MeanL3), and of the (c) tip‐to‐tip distance (TTD) between the cusp tips of the maxillary canines.

All intraoral and extraoral landmarks were placed manually by a single trained examiner according to a predefined protocol. Intraoral measurements comprised the mesiodistal width (measured perpendicular to the tooth's long axis) and cervicoincisal length (from cervical margin to incisal edge) of each maxillary anterior tooth. To account for bilateral asymmetry, mean values were calculated for corresponding tooth pairs, yielding MeanW1–3 and MeanL1–3 for central incisors, lateral incisors, and canines, respectively (Figure 1). The tip‐to‐tip distance (TTD) between the maxillary canine cusps was recorded as an additional parameter.

**FIGURE 2:**
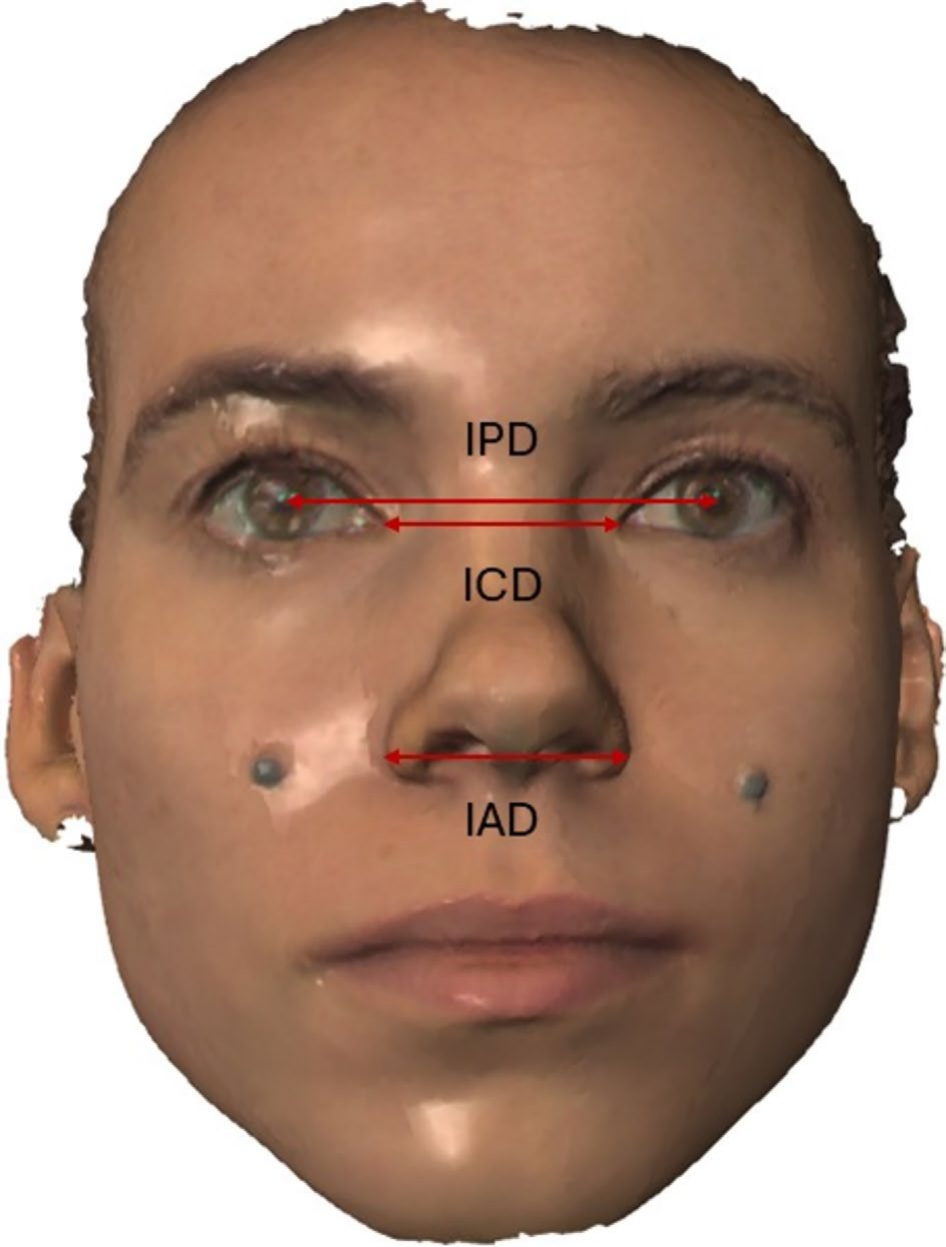
Measurements of the interpupillary distance (IPD), inner canthal distance (ICD), and interalar distance (IAD).

Extraoral measurements included three facial distances: interpupillary distance (IPD), measured center‐to‐center between pupils, inner canthal distance (ICD), between medial canthi, and interalar distance (IAD), measured between the sulcus alaris points bilaterally (Figure 2). The sulcus alaris represents the most lateral point of the curved baseline of each alar wing where it meets the facial skin, forming a distinct anatomical groove. This landmark was chosen over the widest alar point to ensure consistent identification across different nasal morphologies and to minimize measurement variability associated with alar flaring during facial expressions.

To assess intra‐examiner repeatability and point‐specific deviation within the 3D workflow, landmarks were independently re‐placed 10 times on a single test subject prior to the main evaluation. No comparison with manual caliper measurements or cast‐based reference standards was performed.

### 2.4 Statistical Analysis

Statistical analyses were performed using Jamovi version 2.3.26.0 (Jamovi, Sydney, Australia). Descriptive statistics were calculated for all parameters and normal distribution was verified using the Shapiro–Wilk test. Relationships between facial and dental measurements were assessed using Pearson correlation coefficients with the level of significance set at *p* < 0.05. To account for multiple comparisons, false discovery rate (FDR) control was applied using the Benjamini–Hochberg procedure, with adjusted significance set at q < 0.05. Additionally, proportional ratios between measurements were calculated to establish clinical reference values.

## 3 Results

The study included 81 participants (58 females, 23 males) with a mean age of 23.9 years. Fourteen individuals reported no prior orthodontic treatment, while 67 had been treated with fixed appliances, 13 with removable appliances, and 31 with both.

Descriptive statistics for all measured parameters are presented in Table 1. The maxillary central incisors showed the largest dimensions among anterior teeth, with a mean mesiodistal width (MeanW1) of 8.76 ± 0.54 mm and cervicoincisal length (MeanL1) of 10.3 ± 1.01 mm. The canine tip‐to‐tip distance (TTD) measured 34.4 ± 2.21 mm. Extraoral measurements yielded mean values of 33.5 ± 2.89 mm for IAD, 61.1 ± 3.29 mm for IPD, and 37.0 ± 3.39 mm for ICD. All parameters showed a normal distribution according to the Shapiro–Wilk test.

**TABLE 1:** Descriptive statistics of intraoral and extraoral measurements, including mean ± standard deviation (SD), minimum and maximum values, 95% confidence intervals (CI), and results of the Shapiro–Wilk normality test.

Variable	Mean ± SD [mm]	Minimum [mm]	Maximum [mm]	95% CI	Shapiro–Wilk *p*
MeanW1	8.76 ± 0.544	7.38	10.19	8.64–8.88	0.543
MeanW2	6.53 ± 0.599	5.07	7.71	6.40–6.66	0.75
MeanW3	7.59 ± 0.464	6.39	8.52	7.49–7.70	0.698
MeanL1	10.3 ± 1.01	7.86	13.32	10.08–10.52	0.769
MeanL2	8.46 ± 0.939	6.00	10.89	8.25–8.66	0.877
MeanL3	9.42 ± 1.18	5.25	12.53	9.16–9.68	0.355
TTD	34.4 ± 2.21	26.42	39.10	33.86–34.84	0.146
IPD	61.1 ± 3.29	50.40	68.36	60.38–61.84	0.164
ICD	37.0 ± 3.39	27.89	45.57	36.28–37.78	0.112
IAD	33.5 ± 2.89	27.79	40.64	32.85–34.13	0.472

All repeated measurements used to assess intra‐examiner repeatability showed normal distribution. Low variability was observed across all intra‐ and extraoral parameters. Standard deviations were ±0.06 mm for MeanW1 Repeat, ±0.02 mm for MeanL1 Repeat, ±0.12 mm for TTD Repeat, ±0.10 mm for IPD Repeat, ±0.13 mm for ICD Repeat, and ±0.16 mm for IAD Repeat. Bivariate correlation analysis (Table 2) revealed significant associations between TTD and both IAD (*r* = 0.319, *p* = 0.004) and IPD (*r* = 0.303, *p* = 0.006). IAD also correlated significantly with MeanW1 (*r* = 0.254, *p* = 0.022), while MeanW1 and TTD showed strong correlation (*r* = 0.701, *p* < 0.001). Neither IPD nor ICD demonstrated significant bivariate correlations with MeanW1. After FDR correction, significant correlations were found between TTD and IAD (*r* = 0.319, *q =* 0.011), TTD and IPD (*r* = 0.303, *q* = 0.012), MeanW1 and TTD (*r* = 0.701, *q* = 0.004), and MeanW1 and IAD (*r* = 0.254, *q =* 0.035). Other facial‐dental correlations were not significant after correction (*q* > 0.05).

**TABLE 2:** Correlations between intraoral and extraoral measurements with corresponding 95% confidence intervals (CI), *p*‐values, and Benjamini–Hochberg FDR–adjusted *q*‐values *q* (BH‐FDR).

Variable pair	*r*	95% CI	*p*	*q* (BH‐FDR)
MeanW1–TTD	0.701	0.57–0.70	< 0.001	0.004
MeanW1–IAD	0.254	0.03–0.45	0.022	0.035
MeanW1–ICD	−0.037	−0.25–0.18	0.745	0.745
MeanW1–IPD	0.180	−0.04–0.38	0.108	0.144
TTD–IAD	0.319	0.10–0.50	0.004	0.011
TTD–IPD	0.303	0.09–0.49	0.006	0.012
TTD–ICD	0.145	−0.08–0.35	0.196	0.224
IPD–ICD	0.763	0.65–0.84	< 0.001	0.004

Proportional ratios between intraoral dimensions are summarized in Table 3. The mean length‐to‐width ratio was 1.18 ± 0.10 for the maxillary central incisors (MeanL1/MeanW1), 1.30 ± 0.12 for the lateral incisors (MeanL2/MeanW2), and 1.24 ± 0.13 for the canines. Lateral incisors' width represented 74.5% of central incisor width (MeanW2/MeanW1), while canines' width represented 86.8% (MeanW3/MeanW1).

**TABLE 3:** Mean proportional ratios (±SD) between intraoral tooth dimensions and extraoral facial distances, illustrating relative relationships among anterior tooth measurements and facial reference parameters.

	Mean	SD
MeanL1/MeanW1	1.18	±0.101
MeanW1/MeanL1	0.856	±0.073
MeanL2/MeanW2	1.30	±0.122
MeanW2/MeanL2	0.777	±0.074
MeanL3/MeanW3	1.24	±0.132
MeanW3/MeanL3	0.817	±0.099
MeanW2/MeanW1	0.745	±0.057
MeanW3/MeanW1	0.868	±0.054
MeanW1/TTD	0.255	±0.014
MeanW1/IAD	0.263	±0.024
TTD/IAD	1.03	±0.092
TTD/IPD	0.563	±0.04

Proportional ratios between intraoral and extraoral parameters included MeanW1/TTD (0.255 ± 0.014), MeanW1/IAD (0.263 ± 0.024), TTD/IAD (1.03 ± 0.09), and TTD/IPD (0.563 ± 0.04).

## 4 Discussion

The first research hypothesis was supported by a significant association between IAD and the mesiodistal width of the maxillary central incisors, whereas no comparable associations were identified for IPD or ICD. While one previous study reported a relationship between nasal width and maxillary anterior tooth dimensions [3], other investigations failed to demonstrate a significant association with IAD and instead described correlations with IPD or ICD [5, 14, 18, 20].

With respect to the second research hypothesis, significant associations were identified between extraoral facial measurements and TTD. IAD was the only facial parameter significantly associated with both TTD and maxillary central incisor width, whereas IPD demonstrated a relationship with TTD only. The mean TTD observed in the present cohort (34.4 ± 2.21 mm) falls within the range of 34.30–37.44 mm reported in the literature [6, 8, 10, 13]. Absolute IAD values (33.5 ± 2.89 mm) were slightly lower than previously reported ranges of 34.28–41.22 mm [3, 5, 6, 8, 13, 14], a difference likely attributable to variation in landmark definition and measurement technique. The TTD/IAD ratio observed in the present study (1.03 ± 0.092) corresponds to earlier findings based on direct Boley gauge measurements on wax registrations, which reported TTD values approximately 3% greater than IAD [6]. In contrast, studies relying on 2D facial photography failed to demonstrate significant associations between TTD and IAD [13, 14], with one report describing a distinctly lower TTD/IAD ratio of 0.914 [10]. This discrepancy appears largely attributable to methodological differences in facial measurement acquisition. Specifically, the latter study derived a median IAD value of 41.2 mm from frontal 2D photographic analysis [10], considerably higher than the 34.28 mm obtained through direct gauge‐based measurement [6]. These inconsistencies reflect fundamental methodological differences across studies. Earlier investigations commonly defined IAD at the visually widest alar point in 2D photographs, whereas the present 3D protocol referenced the sulcus alaris, an anatomically consistent but narrower landmark that can be reliably identified on facial scans. Differences in dimensionality, landmark definition, and the use of separate acquisition systems for facial and dental measurements therefore represent major sources of variability when comparing absolute IAD values and derived proportions across studies. Although age‐related widening of the nasal base has been suggested and could potentially influence facial‐to‐dental proportions, the current evidence remains inconsistent and does not allow definitive conclusions [9, 13].

In line with the third research hypothesis, proportional relationships between facial measurements and maxillary anterior tooth dimensions were quantifiable within the standardized 3D workflow. The TTD/IAD ratio (1.03 ± 0.092) and the MeanW1/TTD ratio (0.255 ± 0.014) showed low variability, indicating stable proportional relationships when assessed under integrated 3D conditions. Within this framework, facial and dental measurements were evaluated on volumetric datasets within one common coordinate system, improving landmark consistency and reducing projection‐related bias [26]. Methodological consistency in this study was ensured through standardized acquisition conditions, including repeated landmark placement by a single trained examiner with confirmed intra‐examiner reliability, analysis of original exported datasets without mesh smoothing or surface manipulation, and controlled facial expression during scanning. While automated facial landmark detection represents a promising approach to further enhance efficiency and standardization in integrated 3D workflows, the present study focused on controlled landmark definition to characterize proportional relationships and their variability under standardized conditions [26]. The proportional relationships observed were comparable to those previously reported using direct caliper‐based measurements [6], whereas greater deviations were primarily associated with 2D photographic approaches [13]. Comparison with data derived from analogue measurement methods remains challenging, as reproducible caliper‐based assessments on mobile facial soft tissues and consistent identification of the intended measurement location are highly technique‐sensitive and likely more error‐prone than the present approach using 3D facial scanning. Assessment of facial‐to‐dental proportional relationships within an integrated 3D dataset facilitates their direct transfer into digital prosthodontic planning environments that combine facial and intraoral data for virtual tooth setup and restorative design. The contribution of this work therefore lies in demonstrating that these established relationships can be consistently assessed and applied within an anatomically consistent, integrated 3D dataset that allows facial measurements to be acquired with reduced chairside time and less reliance on patient cooperation compared with conventional anthropometric assessment.

Several limitations should be considered. The cohort was intentionally restricted to young, fully dentate adults to allow assessment of relationships between facial measurements and tooth dimensions under morphologically stable conditions, minimizing the influence of age‐related changes and tooth wear. Consequently, the findings cannot be directly generalized to older or edentulous populations. The unequal sex distribution precluded stratified analysis, and ethnicity was not recorded, limiting evaluation of potential population‐specific effects. Furthermore, although intra‐examiner reliability was high, inter‐examiner reliability was not assessed, as all measurements were performed by a single examiner. This single‐examiner approach is consistent with comparable studies on facial and dental proportions, where it is frequently applied to ensure internal consistency. Nonetheless, operator‐dependent landmark placement remains a potential source of variance. Finally, no direct validation against physical reference standards was performed in the present study, though 3D facial imaging systems and intraoral scanners comparable to those applied have been validated against direct anthropometry and conventional measurements in previous investigations [23].

Despite these limitations, the use of advanced 3D imaging, a standardized acquisition and measurement protocol, and a comparatively large sample size support the methodological robustness of the study. Nevertheless, the proportional values reported should be regarded as preliminary reference information intended to support digital treatment planning and require further validation through clinical try‐in procedures and patient‐specific assessment. Future investigations should evaluate these relationships in older and edentulous populations, include balanced sex distributions, and incorporate direct validation against physical reference standards. Moreover, the integration of automated 3D facial landmark detection may further enhance standardization and efficiency in prosthetic workflows, particularly when pre‐extraction records are unavailable or when high esthetic demands require precise restoration of natural tooth proportions and facial harmony.

## 5 Conclusion

Within the limitations of this study, it was concluded that IAD showed the strongest association with maxillary anterior tooth dimensions. The ratio between TTD and IAD was 1.03 ± 0.09, and one quarter of the TTD corresponded to the width of a central incisor (MeanW1/TTD = 0.255 ± 0.014).

## Funding

The authors have nothing to report.

## Conflicts of Interest

The authors declare no conflicts of interest.

## Data Availability

The data that support the findings of this study are available on request from the corresponding author. The data are not publicly available due to privacy or ethical restrictions.
